# Determinants and impact of suboptimal asthma control in Europe: The INTERNATIONA*L* CROSS-SECT*I*ON*A*L AND LONG*I*TUDINAL ASSESSMENT ON A*S*THMA C*ON*TROL (LIAISON) study

**DOI:** 10.1186/s12931-016-0374-z

**Published:** 2016-05-14

**Authors:** Fulvio Braido, Guy Brusselle, Daniele Guastalla, Eleonora Ingrassia, Gabriele Nicolini, David Price, Nicolas Roche, Joan B. Soriano, Heinrich Worth, on behalf of the LIAISON Study Group

**Affiliations:** Allergy and Respiratory Diseases Clinic, University of Genoa, IRCCS-AOU San Martino, Genoa, Italy; Department of Respiratory Medicine, Ghent University Hospital, Ghent, Belgium; Departments of Epidemiology and Respiratory Medicine, Erasmus MC, Rotterdam, The Netherlands; Chiesi Farmaceutici S.p.A., Via Palermo 26/A, Parma, 43122 Italy; Centre of Academic Primary Care, University of Aberdeen, Aberdeen, UK; Service de Pneumologie et Réanimation, Hôtel-Dieu, Groupe Hospitalier Cochin-Broca-Hôtel-Dieu, Assistance Publique-Hôpitaux de Paris, Université Paris Descartes, Paris, France; Instituto de Investigación Hospital Universitario de la Princesa (IISP), Universidad Autónonoma de Madrid, Madrid, Spain; Facharztforum Fürth, Fürth, Germany

## Abstract

**Background:**

According to the Global Initiative of Asthma, the aim of asthma treatment is to gain and maintain control. In the INTERNATIONA*L* CROSS-SECT*I*ON*A*L AND LONG*I*TUDINAL ASSESSMENT ON A*S*THMA C*ON*TROL (LIAISON) study, we evaluated the level of asthma control and quality of life (QoL), as well as their determinants and impact in a population consulting specialist settings.

**Methods:**

LIAISON is a prospective, multicentre, observational study with a cross-sectional and a 12-month longitudinal phase. Adults with an asthma diagnosis since at least 6 months, receiving the same asthma treatment in the 4 weeks before enrolment were included. Asthma control was assessed with the 6-item Asthma Control Questionnaire (ACQ) and QoL with the MiniAsthma Quality of Life Questionnaire (MiniAQLQ).

**Results:**

Overall, 8111 asthmatic patients were enrolled in 12 European countries. Asthma control was suboptimal in 56.5 % of patients and it was associated with poorer asthma-related QoL, higher risk of exacerbations and greater consumption of healthcare resources.

Variables associated with suboptimal control were age, gender, obesity, smoking and comorbidities. Major determinants of poor asthma control were seasonal worsening and persisting exposure to allergens/irritants/triggers, followed by treatment-related issues.

**Conclusions:**

The cross-sectional phase results confirm that suboptimal control is frequent and has a high individual and economic impact.

**Trial registration:**

The clinicaltrials.gov identifier is NCT01567280.

**Electronic supplementary material:**

The online version of this article (doi:10.1186/s12931-016-0374-z) contains supplementary material, which is available to authorized users.

## Background

The main goals of asthma management are to control symptoms, prevent exacerbations, improve and maintain lung function while minimizing side-effects of asthma medications. Improvements in asthma control do not only impact the patients’ daily life but are also associated with a reduced risk of exacerbations and lung function impairment. Additionally, gaining and maintaining asthma control is expected to be cost-effective by reducing the overall cost of asthma care and indirect costs related to decreased productivity. However, in spite of the widespread availability of therapies reported as highly effective in randomised controlled trials [1], variable levels of asthma control have been shown in several “real-life” studies using well-validated self-assessment questionnaires, such as the Asthma Control Questionnaire (ACQ) [2] and the Asthma Control Test (ACT) [3]. In a recent study performed in the Netherlands in 200 adult asthma patients, the percentage of patients with partly controlled or uncontrolled asthma was 35.5 % and 27.0 % respectively, as measured by the ACQ [4]. In contrast, observational studies performed in Italy reported about 35 % of suboptimal asthma control [5, 6]. European-wide, population-based surveys reported prevalence rates of unsatisfactory asthma control levels ranging from 56.6 % [7] to 80.0 % [8, 9].

The discrepancies observed between the results of randomised controlled trials and observational studies are due to the different methodologies applied, the former requiring more restrictive eligibility criteria for the identification of a highly selected population and closer control of single study subjects. In contrast, “real-life” studies comprise larger and more diverse patient populations with/without comorbidities, different levels of treatment adherence and with different approaches in the disease management depending on various factors, like, among others, the qualification of the treating physician, the availability of healthcare resources, or the patient’s education to a healthy lifestyle. Moreover, the use of different definitions of asthma control and different tools to measure it makes it difficult to compare different results coming from studies on asthma control [10].

The “INTERNATIONA*L* CROSS-SECT*I*ON*A*L AND LONG*I*TUDINAL ASSESSMENT ON A*S*THMA C*ON*TROL” (LIAISON) study [11] was carried out to evaluate the determinants and impact of suboptimal asthma control and related quality of life (QoL).

## Methods

### Study design

LIAISON is a multinational, prospective, observational cohort study designed to provide cross-sectional and longitudinal information on asthma control. The study was performed in Austria, Belgium, France, Germany, Greece, Hungary, Italy, the Netherlands, Poland, Spain, Turkey and the United Kingdom and recruited patients between May 2012 and October 2013. In this first report we describe data from the cross-sectional phase of the study, which aimed at evaluating: (i) the proportion of patients with controlled, partly controlled and uncontrolled asthma; (ii) health-related Quality of Life (HRQoL) and its relation to the level of asthma control; and (iii) factors associated with suboptimal asthma control.

The study was conducted in accordance with the current International Conference on Harmonization Good Clinical Practice Guidelines, the World Medical Association Declaration of Helsinki (59th WMA General Assembly, Seoul, October 2008) and all other applicable local laws and regulations. Patients gave written informed consent and study approval was obtained from all relevant local ethics committees and national regulatory authorities.

### Study subjects

Male and female adult patients (smokers and non-smokers) with a GINA-compliant diagnosis of asthma for at least 6 months and treated with the same anti-asthmatic drugs in the last 4 weeks before enrolment were eligible for study participation. Patients suffering from critical conditions and illnesses that might have interfered (according to the doctors’ judgment) with the study purposes were excluded.

### Collected data and measurements

The following items were collected and their relations to asthma control were analysed: demographic characteristics (age, gender, body mass index, education level, living environment, employment status, occupational exposure to allergens/irritants/triggers, smoking habits, allergies, concomitant diseases relevant to respiratory tract and outpatients visits), characteristics of asthma (age and severity at diagnosis, qualification of the physician and investigations made for the diagnosis of asthma, current severity of asthma symptoms, need of rescue medication and participation in asthma education programs), the reasons for poor control from the patient’s and the physician’s perspective (according to their subjective judgment; objective evidence were not required), the anti-asthmatic treatments and the propensity to adhere to therapy.

Self-administered tools were used for the assessment of (i) asthma control (6-item ACQ), (ii) HRQoL (MiniAQLQ) and (iii) propensity to adhere to therapy (4-item Morisky Medication Adherence Scale, MMAS-4).

Number of spirometries, Peak Expiratory Flow (PEF), Forced Expiratory Volume in 1 s (FEV_1_), Forced Vital Capacity (FVC) and the ratio FEV_1_/FVC values related to the last 12 weeks before the cross-sectional visit, were collected and analysed, when available.

Healthcare resources utilization [emergency room (ER) admittance, hospitalizations, days missed from work due to asthma and support received by family] and severe exacerbations (defined as deterioration of asthma resulting in hospitalization or ER treatment or need of systemic steroids for more than 3 days) in the last 12 months before the cross-sectional visit, were collected and analysed.

### Statistical analysis

The prevalence of patients with controlled, partly controlled and uncontrolled asthma was calculated as the proportion of patients with an ACQ score of ≤0.75, >0.75–1.5 and ≥1.5, respectively, over the total of evaluable patients at the cross-sectional phase visit.

Descriptive summary statistics [mean, standard deviation (SD)] were applied for the MiniAQLQ score and other continuous variables. For the ACQ score and other categorical variables, frequencies and percentages were provided. The Kruskal-Wallis test was used to compare continuous variables between the different asthma control level groups. For the comparisons of categorical variables, the Chi-square test was performed. The description of the smoking habit considered current smokers, ex-smokers and never-smokers as separate groups of patients.

The sample size was estimated in relation to the expected proportion of patients with controlled asthma at the cross-sectional phase visit (approximately 37.5 %) [4] and patients with partly controlled/uncontrolled asthma at the cross-sectional phase visit reaching asthma control after 12 months (approximately 45 %) [12]. Considering a drop-out rate of 20 %, an initial patient population of 8150 subjects was calculated in order to have the expected number of 4075 patients evaluable for the assessment of the expected 45 % of patients reaching asthma control at the end of the longitudinal phase with a precision of ± 1.5 % [two-sided 95 % confidence interval (CI)].

Considering the expected number of patients reaching asthma control at the end of the longitudinal phase, the distance from the boundaries of the two-sided 95 % CI to the point estimate is 0.055 units for the MiniAQLQ used for the evaluation of QoL, based on a SD of 1.21 units [13].

A multiple logistic regression model was built using stepwise procedures to assess the variables independently associated with asthma control and HRQoL.

## Results

### Description of the study population

Out of 8178 patients attending 153 outpatient hospitals or General Practice clinics, 8111 were considered eligible for the analysis.

The patients’ distribution by country in descending order was: Italy 13.6 % (*n* = 1,101), Germany 13.2 % (*n* = 1,071), Spain 12.8 % (*n* = 1,041), Poland 12.8 % (*n* = 1,037), France 10.5 % (*n* = 854), Hungary 10.5 % (*n* = 851), Turkey 5.4 % (*n* = 439), the United Kingdom 4.9 % (*n* = 399), Belgium 4.7 % (*n* = 382), Greece 4.2 % (*n* = 339), Austria 4.0 % (*n* = 324) and the Netherlands 3.4 % (*n* = 274).

Main patients’ characteristics are depicted in Table 1.

**Table 1:** Demographic and clinical characteristics of participants

	Controlled *n* = 3526 (43.5)	Partly controlled *n* = 1462 (18.0)	Uncontrolled *n* = 3123 (38.5)	Overall *p*-value*	Overall population *n* = 8111 (100.0)
Age [years], mean (SD)	49 (16)	49 (16)	52 (15)	<.001	50 (16)
Age categories (years), *n* (%)				<.001	
≤ 39	991 (30.1)	407 (30.0)	572 (21.9)		1970 (27.1)
40–64	1612 (49.0)	694 (51.1)	1519 (58.2)		3825 (52.7)
≥ 65	688 (20.9)	257 (18.9)	518 (19.9)		1463 (20.2)
Gender, *n* (%)					
Female	2207 (62.8)	996 (68.3)	2184 (70.1)	<.001	5387 (66.6)
Body mass index categories, *n* (%)				<.001	
Normal weight (≥18.5 and <25 kg/m^2^)	1469 (41.7)	545 (37.3)	1044 (33.4)		3058 (37.7)
Obese (≥30 kg/m^2^)	763 (21.7)	384 (26.3)	968 (31.0)		2115 (26.1)
Overweight (≥25 and <30 kg/m^2^)	1238 (35.1)	505 (34.5)	1069 (34.2)		2812 (34.7)
Underweight (<18.5 kg/m^2^)	53 (1.5)	28 (1.9)	42 (1.3)		123 (1.5)
Living environment, *n* (%)				0.038	
Rural	871 (26.8)	412 (30.0)	792 (27.1)		2075 (27.5)
Urban	2374 (73.2)	960 (70.0)	2131 (72.9)		5465 (72.5)
Education, *n* (%)				<.001	
None	54 (1.8)	13 (1.0)	52 (1.9)		119 (1.7)
Primary/middle school	1122 (36.9)	528 (39.9)	1235 (45.7)		2885 (40.8)
High school	1179 (38.8)	461 (34.8)	890 (32.9)		2530 (35.8)
Graduate/Post Graduate	685 (22.5)	321 (24.3)	528 (19.5)		1534 (21.7)
Employment status, *n* (%)				<.001	
Employed	1811 (56.8)	734 (54.0)	1310 (45.1)		3855 (51.8)
Unemployed/housewife/student	602 (18.9)	263 (19.3)	553 (19.1)		1418 (19.0)
Retired/Unable to work	773 (24.3)	363 (26.7)	1039 (35.8)		2175 (29.2)
Occupational exposure to allergens/irritants among employed, *n* (%)	581 (32.1)	294 (40.1)	542 (41.4)	0.008	1417 (36.8)
Smoking habits, *n* (%)				<.001	
Never-smoker	2312 (66.6)	913 (63.8)	1966 (63.8)		5191 (65.0)
Current Smoker	314 (9.0)	182 (12.7)	425 (13.8)		921 (11.5)
Ex-Smoker^a^	846 (24.4)	337 (23.5)	691 (22.4)		1874 (23.5)
Smoking history pack years^b^, mean (SD)	14 (14)	15 (14)	16 (16)	<.001	15 (15)
Pack years ≥10, *n* (%)	588 (51.9)	280 (55.9)	641 (58.5)	0.007	1509 (55.3)
Allergies, *n* (%)				0.059	
Yes	2327 (68.6)	953 (67.3)	1992 (65.8)		5272 (67.3)
No	1065 (31.4)	463 (32.7)	1035 (34.2)		2563 (32.7)
Frequent allergies^c^					
Inhalants, *n* (%)	2148 (92.3)	861 (90.3)	1783 (89.5)	0.005	4792 (90.9)
Drugs, *n* (%)	247 (10.6)	115 (12.1)	312 (15.7)	<.001	674 (12.8)
Foods, *n* (%)	219 (9.4)	98 (10.3)	207 (10.4)	0.520	524 (9.9)
Comorbidities relevant to respiratory tract^d^, *n* (%)					
Allergic rhinitis	976 (55.8)	452 (57.6)	860 (50.3)	<.001	2288 (53.9)
Hypertension	508 (29.0)	247 (31.5)	629 (36.8)	<.001	1384 (32.6)
Gastro − esophageal reflux	351 (20.1)	181 (23.1)	500 (29.2)	<.001	1032 (24.3)
Chronic rhinosinusitis	259 (14.8)	96 (12.2)	276 (16.1)	0.040	631 (14.9)
Psychological disturbances	145 (8.3)	95 (12.1)	333 (19.5)	<.001	573 (13.5)
Respiratory infections	105 (6.0)	81 (10.3)	288 (16.8)	<.001	474 (11.2)
Nasal polyposis	165 (9.4)	54 (6.9)	150 (8.8)	0.107	369 (8.7)
Obstruction Sleep Apnea Syndrome	74 (4.2)	33 (4.2)	92 (5.4)	0.216	199 (4.7)
Congestive heart failure	27 (1.5)	27 (3.4)	113 (6.6)	<.001	167 (3.9)
Outpatients visits^e^					
Mean (SD)	5.3 (5.6)	6.9 (6.5)	8.3 (7.7)	<001	6.7 (6.8)
Patients with at least one visit, *n* (%)	3353 (95.1)	1399 (95.7)	2996 (95.9)	0.241	7748 (95.5)

The percentages have been calculated based on the number of available data for each variable

*n* number of patients, *SD* standard deviation

*p-value based on Chi-square for categorical variables and Kruskal-Wallis test for quantitative variables

^a^Patients who stopped smoking at least one year prior to study start

^b^Refers to current smokers and ex-smokers

^c^The percentages are calculated based on the number of patients with allergies

^d^Patients could have more than one comorbidity

^e^Including visits to respiratory physician, allergologist, other specialist and general practitioner in the last 12 months

### Asthma control and quality of life

Among 8111 patients, 43.5 % (*n* = 3,526) showed controlled asthma, 18.0 % (*n* = 1,462) had partly controlled asthma and 38.5 % (*n* = 3,123) uncontrolled asthma. The mean (SD) of ACQ score was 0.27 (0.24), 1.07 (0.19) and 2.48 (0.80) in patients with controlled, partly controlled and uncontrolled asthma, respectively.

**Fig. 1:**
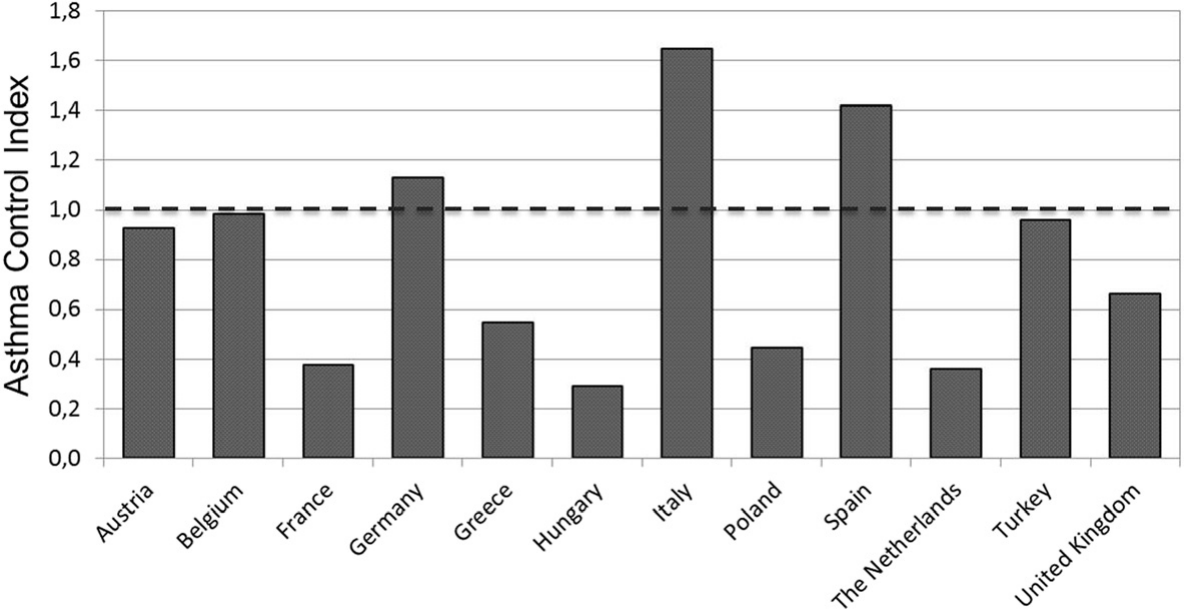
Asthma Control Index among countries of the LIAISON study. The index was computed as the ratio of patients with controlled asthma (6-item ACQ < 0.75) to patients with not well-controlled asthma (6-item ACQ ≤ 0.75). Asthma Control Index >1: greater proportion of patients with controlled asthma. Asthma Control Index <1: greater proportion of patients with not well-controlled asthma

Regarding Asthma Control Index by country, patients in Italy and Spain had greater levels of asthma control, whereas the lowest rates were observed in Hungary, The Netherlands and France (Fig. 1).

Several demographic and clinical differences were observed among patients of the three asthma control levels, especially in the comparison of patients with controlled vs uncontrolled asthma (Table 1).

Table 2 shows the relationship between the study variables and the control of asthma. Obese patients were significantly more likely to have partly controlled/uncontrolled asthma compared to patients with normal weight. The same was true for female patients vs male patients as well as current smokers vs never-smokers. As regards concomitant diseases, the risk of having partly controlled/uncontrolled asthma was higher for patients reporting respiratory infections, congestive heart failure and psychological disturbances.

**Table 2:** Association between partly controlled/uncontrolled asthma and demographics and comorbid conditions

Variable	Odd ratio	95 % CI	*p*-value
Gender			
Male vs. Female	0.7286	0.6542, 0.8115	<.0001
Age [years]			
18–39 vs. 40–64	0.8563	0.7576, 0.9678	0.0130
Over 65 vs. 40–64	0.7667	0.6707, 0.8766	0.0001
BMI			
Overweight vs. Normal weight	1.2823	1.1365, 1.4467	<.0001
Obese vs. Normal weight	1.7139	1.4985, 1.9603	<.0001
Smoking habits			
Current Smoker vs. Never smoker	1.5852	1.3474, 1.8649	<.0001
Environment			
Rural vs. Urban	1.2419	1.1094, 1.3902	0.0002
Gastro-esophageal reflux			
Yes vs. no	1.2480	1.0623, 1.4661	0.0070
Psychological disturbances			
Yes vs. no	1.8714	1.5087, 2.3212	<.0001
Respiratory infections			
Yes vs. no	2.4152	1.8992, 3.0715	<.0001
Congestive heart failure			
Yes vs. no	3.4972	2.2226, 5.5027	<.0001

*BMI* body mass index, *CI* confidence interval

The reference group is made of controlled asthmatics. Subjects with available data on variables included in the model were considered (*n* = 6421)

Variables included in the logistic regression model were identified after stepwise selection

For each stepwise selection, it was considered 0.05 as significance level for entering and for staying in the model

**Fig. 2:**
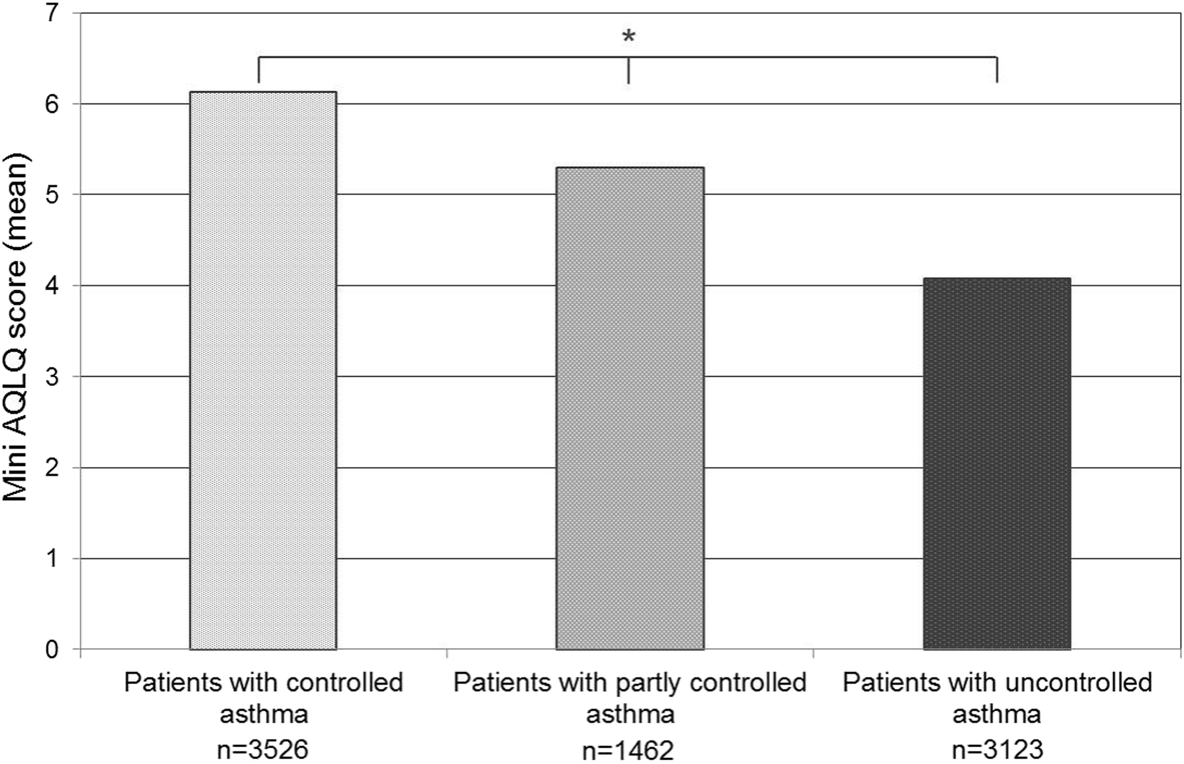
Mean overall score of the Mini Asthma Quality of Life Questionnaire by asthma control level. *Overall *p*-value < .001

Better QoL was reported in patients with controlled asthma and a positive correlation between the level of asthma control and the QoL level was shown (Fig. 2 and Additional file 1: Figure S1, respectively).

A higher score of MiniAQLQ was positively related to younger age (18–39 years) as well as higher age (over 65 years) and male gender, while obesity was negatively related to the MiniAQLQ score. As observed for asthma control, a lower MiniAQLQ score was associated with the presence of congestive heart failure, respiratory infections and psychological disturbances (Table 3).

**Table 3:** Relationship between quality of life by MiniAQLQ and demographics and comorbid conditions

Variable	Beta	95 % CI	*p*-value
Gender			
Male vs. Female	0.2638	0.1976, 0.3299	<.0001
Age [years]			
18–39 vs. 40–64	0.2786	(0.2027, 0.3545)	<.0001
Over 65 vs. 40–64	0.1677	(0.0861, 0.2493)	<.0001
BMI			
Obese vs. Normal weight	−0.2346	(−0.3055, −0.1636)	<.0001
Ethnicity			
Asian vs. White	−0.8009	−1.3949, −0.2069	0.0082
Smoking habits			
Ex-Smoker vs. never-smoker	0.0991	(0.0264, 0.1717)	0.0075
Education			
Graduate/Post Graduate vs. Primary or middle school	0.3355	(0.2520, 0.4189)	<.0001
High school vs. Primary or middle school	0.2008	(0.1307, 0.2708)	<.0001
Allergy to Inhalants			
Yes vs. no	−0.0942	(−0.1597, −0.0288)	0.0048
Psychological disturbances			
Yes vs. no	−0.5340	(−0.6497, −0.4184)	<.0001
Gastro-esophageal reflux			
Yes vs. no	−0.1626	(−0.2565, −0.0688)	0.0007
Respiratory infections			
Yes vs. no	−0.5875	(−0.7151, −0.4599)	<.0001
Congestive heart failure			
Yes vs. no	−0.6949	(−0.8963, −0.4935)	<.0001

*BMI* body mass index, *CI* confidence interval

Subjects with available data on variables included in the model were considered (*n* = 5618)

Variables included in the logistic regression model were identified after stepwise selection. For each stepwise selection, it was considered 0.05 as significance level for entering and for staying in the model

### Characteristics of asthma

The diagnosis of asthma was made by a respiratory physician in 66.6 % of cases, while the remaining diagnoses were confirmed by general practitioners (16.9 %) and other, unspecified specialists (9.5 %). Such information was not available for 7 % of participants. Investigations performed as part of the diagnostic workup included spirometry (79.8 % of patients), allergy tests (53.1 %), chest x-ray (45.8 %), PEF (26.6 %), IgE measurement (20.7 %), bronchial provocation (17.1 %) and FENO (5.6 %). Asthma was predominantly persistent at diagnosis (69.5 %) and was rated severe in 23.6 % of uncontrolled patients (vs 12.7 % among controlled patients).

Daily occurrence of asthma symptoms during the last week before the visit was reported in 37.3 % of uncontrolled patients and need of daily rescue short-acting beta agonist (SABA) use in 34.7 % of uncontrolled patients (vs 2.0 % and 2.7 % of patients with controlled asthma).

The percentage of patients reporting severe exacerbations in the last 12 months before the visit was lower in patients with controlled asthma compared to those with partly controlled (*p* = 0.019) and uncontrolled disease (*p* < 0.001). One severe exacerbation was reported by 8.9 % of controlled-asthma patients and 13.6 % of uncontrolled-asthma patients. A higher proportion of uncontrolled patients suffered from more than two severe exacerbations when compared to patients with controlled asthma (6.9 % vs 1.5 %).

We found no difference in the percentage of patients participating in an asthma education program across the three groups (Additional file 1: Table S1). By contrast, a difference was detected in investigators’ participation in an asthma education program (46.6 % of controlled-group vs 35.8 % of uncontrolled group).

The percentage of patients who underwent spirometry in the last 12 weeks before the cross-sectional visit was high (73.3 %), and higher in controlled vs uncontrolled patients (75.0 % vs 70.6 %; *p* < 0.001). Lung function was poorer in uncontrolled than in controlled patients (Additional file 1: Table S2).

### Factors associated with poor control

The main reasons for poor asthma control were ranked with similar importance by patients and doctors, though with different percentages (multiple answers were permitted). Seasonal worsening and continued exposure to allergens/irritants/triggers were identified most frequently as the main reason for poor control with kappa coefficients indicating a substantial agreement between doctors and patients. Interestingly, active smoking was considered as a possible reason for poor control by only 10.1 % of doctors and 7.4 % of patients.

If the treatment-related aspects (poor adherence, inadequate therapy, poor efficacy, inadequate inhalation technique, poor tolerability) are cumulated, treatment as a whole can be upgraded as the most important factor for poor asthma control representing 60.0 % of the reasons expressed by doctors and 42.7 % of those expressed by patients (Table 4).

**Table 4:** Reasons for poor asthma control, according to patients’ and doctors’ perspective

Reasons for poor control^a^: *n* = 4585	Patients’ perspective *n* (%)	Doctors’ perspective *n* (%)	Kappa coefficient^b^
Seasonal worsening Continued exposure to allergens/irritants/triggers Comorbidities Poor adherence to therapy Inadequate therapy Poor efficacy of therapy Active smoking Depression Passive smoking Inadequate inhalation technique Poor patient-physician communication Poor tolerability of therapy Co-administration of drugs Incorrect diagnosis	1848 (40.3) 1148 (25.0) 769 (16.8) 507 (11.1) 571 (12.5) 675 (14.7) 339 (7.4) 388 (8.5) 421 (9.2) 104 (2.3) 78 (1.7) 97 (2.1) 70 (1.5) 57 (1.2)	1756 (38.3) 1270 (27.7) 1028 (22.4) 965 (21.0) 868 (18.9) 622 (13.6) 462 (10.1) 453 (9.9) 319 (7.0) 195 (4.3) 197 (4.3) 100 (2.2) 68 (1.5) 40 (0.9)	0.72 0.73 0.68 0.47 0.56 0.53 0.81 0.67 0.64 0.39 0.27 0.44 0.55 0.30

*N* number of patients

^a^More than one reason could be indicated

^b^Kappa agreement interpretation: <0: poor, 0.01–0.20: slight, 0.21–0.40: fair, 0.41–0.60: moderate, 0.61–0.80: good, 0.81–1.00; very good

### Anti-asthma therapies and propensity to adhere

Fixed combinations of an inhaled corticosteroid (ICS) and a long-acting beta agonist (LABA) were the most frequently used current maintenance anti-asthmatic therapies (70.5 % of patients) followed by leukotriene receptor antagonists (24.9 %) and ICS (24.4 %).

Among patients treated with ICS/LABA fixed combinations, 35.0 % were on monotherapy, showing a percentage of patients with controlled asthma of 50.6 %.

The mean MMAS-4 score was similar in the three subgroups of patients with an average rating = 1, corresponding to an intermediate level of treatment adherence. However, a higher rate of low adherence (score 3–4) was present in patients with uncontrolled asthma compared to controlled patients (16.3 % vs 13.0 %). No significant interaction was found between the asthma control level and the non-adherence categories (*p* = 0.398; Additional file 1: Table S3).

### Healthcare resource consumption

The rate of patients who required hospitalization or admitted to an ER was significantly lower in patients with controlled asthma than in those with partly controlled and uncontrolled asthma, even if the total number of events reported in the last 12 months was quite low (4.6 % of patients were hospitalized and 5.4 % admitted to the ER). Significant differences among the three level of control were detected considering the number of days missed from work and the support received by the family (Additional file 1: Table S4).

Differences between patients with controlled asthma and those not-well controlled are shown by country in Additional file 1: Table S5.

## Discussion

### Main findings

The results of the cross-sectional phase of the LIAISON study show that a good control of asthma is achieved in less than 50 % of patients attending specialist settings. Suboptimal control was associated with poorer QoL, a higher risk of exacerbations and greater consumption of healthcare resources. Suboptimal control of asthma has also been reported in numerous previous studies [4, 7, 8, 14, 15] based on different measures of asthma control, sometimes self-reported by the patient.

Variables associated with control status were age, gender, obesity, smoking, psychological status, history of respiratory infections and congestive heart failure. The same variables were associated with asthma-related QoL, together with education level and allergies. In agreement with previous findings [7], our results confirm that the control of asthma is worse in women than in men, and that the female gender increases the probability of uncontrolled asthma. Moreover, obesity and current smoking were confirmed to be associated with worse control of the disease. In contrast with the same study, our data don’t show a relation between increased age and suboptimal control, which may relate to differences in population characteristics (e.g., regarding comorbidities) and care settings (the study by Demoly et al. was based on a self-reported patient database).

### General determinants of poor control

According to patients’ and doctors’ perspectives, major determinants of poor asthma control were related to environmental aspects followed by treatment-related issues. The main reasons for poor asthma control were reported to be related to seasonal worsening and continued exposure to allergens/irritants/triggers, as well as comorbidities relevant to respiratory tract. Depression, already reported as an independent risk factor for poor asthma control [16], was indicated as a specific reason of uncontrolled asthma in 8.5 % of the LIAISON patients as well as in 9.9 % of doctors. Although about 11.5 % of patients were current smokers, only 7.4 % of patients (and 10.1 % of doctors) considered the active smoking as a possible cause of asthma worsening. Similarly, passive smoking was recognized as a possible reason for poor control by less than 10 % of patients and doctors.

Treatment-related factors are crucial for the control of the disease, representing 60.0 % and 42.6 % of all reasons for poor control (as expressed by doctors and patients, respectively), as a whole. The risk of non-adherence was low in the overall study population as well as in uncontrolled subjects (16.3 %). Two times more physicians than patients identified poor adherence to therapy as a potentially critical factor for poor asthma control, which can relate to different perspectives and understanding about the disease by the patients compared to the physicians [17]. Although patient-physician communication did not seem to represent a discriminant factor for asthma control in our study, good communication is important to establish effective collaboration and helps solving problems of proper inhalation technique like difficulties in device handling [14, 18].

These considerations point to the interrelation of various factors impacting on asthma control such as the patients’ underestimation of their symptoms [15] and the complexity of disease management in the presence of comorbidities and environmental/seasonal factors.

The fixed combination of inhaled corticosteroids and long-acting beta-agonists was the most widely used therapy in the LIAISON study. Although randomised controlled clinical trials have demonstrated high efficacy and tolerability of the available anti-asthma medications, real-life data like the LIAISON study, still underline that medications alone are not sufficient to obtain control if other factors are not sufficiently taken into consideration such as the understanding of the particular characteristics of each patient and a careful follow-up of the course of disease.

### Impact of poor asthma control

Importantly, the achievement of good asthma control influences the level of QoL, as shown in our study based on the MiniAQLQ, as well as in other studies using generic QoL tools [5, 19]. Other studies found that suboptimal control also interacts with activity limitations, work productivity or the frequency of emergency visits [15, 19]. Previous findings showed that both a history of past asthma exacerbations and poor asthma control can increase the risk of future asthma exacerbations [20, 21]. In this study, we found a difference in severe exacerbations across the asthma control levels but it has to be taken into account that 12.6 % of patients with good asthma control reported a history of severe exacerbation over the previous year. Exacerbations, hospitalizations or ER admittance were lower in controlled patients, confirming the importance of asthma control also in terms of healthcare resource consumption [5, 15, 19, 22]. Notably, a greater proportion of controlled patients underwent a spirometry test in the last 12 weeks before the cross-sectional visit as compared to uncontrolled patients.

### Strengths and limitations

The data presented in this manuscript were analysed for the whole study population but what should be taken into account is that the management of patients with asthma as well as the care settings are different among the countries involved in the study. The differences among countries involved is probably reflective of the point of care for patient recruitment i.e., primary or secondary consult. Also, the nature of these consults, whether routine or otherwise.

These populations are not a representative cohort and larger studies are needed to address those factors. The focus of this manuscript is to show the impact of asthma control on individual quality of life as well as healthcare resource utilization.

Some of the data analysed (comorbidities, severe exacerbations, emergency department visits or hospitalizations) were reported by patients and could not be directly verified.

As an additional limitation, some comorbidities such as rhinitis could impact the results. Unfortunately, chronic obstructive pulmonary disease (COPD) was not included in the recorded comorbidities, so it is not possible to exclude that some patients had this concomitant diagnosis.

## Conclusion

The LIAISON study is one of the largest observational studies on characteristics and management of asthmatics in Europe based on patient reported outcomes. The results of the cross-sectional phase confirm that many patients with asthma are not well-controlled in real-life despite the wide availability of highly efficacious treatments. Poorer control of asthma is associated with a worse QoL, an increased risk of exacerbations and more consumption of healthcare resources. Many reasons contribute to poorly controlled asthma, both clinical (i.e., comorbidities) and behavioural (i.e., smoking or non-adherence). Their identification could affect the achievement of asthma control, since most risk factors are modifiable.

Moreover, we expect data analysis of the longitudinal phase of LIAISON; characterizing partly controlled/uncontrolled asthma over a one-year follow-up in the near future, to be complementary and highly informative.

## Additional file

Additional file 1: Figure S1. Scatter plot of ACQ and miniAQLQ scores. **Table S1.** Characteristics of asthma. **Table S2.** Lung function. **Table S3.** Propensity to adhere to therapy. **Table S4.** Healthcare resources consumption. **Table S5.** Healthcare and economic resources consumption by country. (DOCX 155 kb)
