# Supplementary material for: Determinants and impact of suboptimal asthma control in Europe: The INTERNATIONAL CROSS-SECTIONAL AND LONGITUDINAL ASSESSMENT ON ASTHMA CONTROL (LIAISON) study
Source: Respir Res. 2016 May 14;17:51. doi: 10.1186/s12931-016-0374-z (PMC4894377; doi:10.1186/s12931-016-0374-z)
Supplement: Additional file 1: Figure S1. — Scatter plot of ACQ and miniAQLQ scores. Table S1. Characteristics of asthma. Table S2. Lung function. Table S3. Propensity to adhere to therapy. Table S4. Healthcare resources consumption. Table S5. Healthcare and economic resources consumption by country. (DOCX 155 kb) [file 12931_2016_374_MOESM1_ESM.docx]

**Figure S1:** Scatter plot of ACQ and miniAQLQ scores


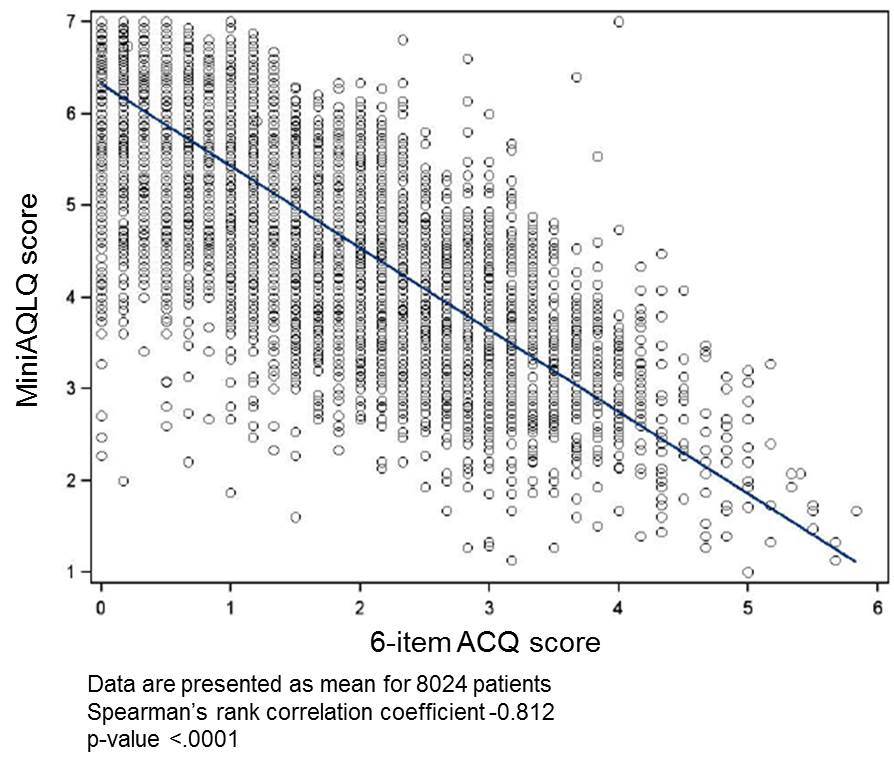


**Table S1:** Characteristics of asthma

|  | **Controlled**  **n=3526 (43.5)** | **Partly controlled**  **n=1462 (18.0)** | **Uncontrolled**  **n=3123 (38.5)** | **Overall p-value*** | **Overall population**  **n=8111 (100.0)** |
| --- | --- | --- | --- | --- | --- |
| **Age at diagnosis [years], n (%)**  Under 18  18-39  40-64  ≥65 | 675 (21.6)  1156 (37.0)  1111 (35.5)  184 (5.9) | 299 (23.0)  475 (36.5)  476 (36.5)  52 (4.0) | 618 (21.9)  912 (32.3)  1145 (40.6)  147 (5.2) | <.001 | 1592 (21.9)  2543 (35.1)  2732 (37.7)  383 (5.3) |
| **Diagnosis of childhood asthma^a^, n (%)** | 476 (15.2) | 226 (17.4) | 434 (15.4) | 0.178 | 1136 (15.7) |
| **Diagnosis made by:**  Respiratory Physician, n (%)  General Practitioner, n (%)  Other specialist, n (%) | 2438 (73.6)  551 (16.6)  323 (9.8) | 931 (68.7)  263 (19.4)  161 (11.9) | 2032 (70.7)  558 (19.5)  285 (9.9) | 0.003 | 5401 (66.6)  1372 (16.9)  769 (9.5) |
| **Investigations made for diagnosis^b^:**  Spirometry, n (%)  Skin test, n (%)  Chest X-ray, n (%)  Peak expiratory flow, n (%)  Challenge test, n (%)  IgE measurements [RAST] , n (%)  FeNO, n (%)  Other, n (%) | 2817 (79.9)  1863 (52.8)  1481 (42.0)  954 (27.1)  783 (22.2)  728 (20.6)  242 (6.9)  115 (3.3) | 1139 (77.9)  742 (50.8)  658 (45.0)  435 (29.8)  237 (16.2)  291 (19.9)  91 (6.2)  43 (2.9) | 2517 (80.6)  1699 (54.4)  1572 (50.3)  769 (24.6)  366 (11.7)  663 (21.2)  120 (3.8)  139 (4.5) | 0.100  0.066  <.001  <.001  <.001  0.580  <.001  - | 6473 (79.8)  4304 (53.1)  3711 (45.8)  2158 (26.6)  1386 (17.1)  1682 (20.7)  453 (5.6)  297 (3.7) |
| **Severity at diagnosis (GINA):**   - intermittent, n (%) - persistent, n (%)   mild  moderate  severe | 973 (33.2)  1959 (66.8)  831 (42.4)  879 (44.9)  249 (12.7) | 390 (32.6)  808 (67.4)  227 (28.1)  452 (55.9)  129 (16.0) | 684 (26.4)  1906 (73.6)  373 (19.6)  1084 (56.9)  449 (23.6) | <.001  <.001 | 2047 (30.5)  4673 (69.5)  1431 (30.6)  2415 (51.7)  827 (17.7) |
| **Current^c^ worst frequency of asthma symptoms:**  None  ≤ 2 days/week  > 2 days/week  Everyday | 2019 (57.3)  1304 (37.0)  131 (3.7)  70 (2.0) | 206 (14.1)  718 (49.1)  367 (25.1)  171 (11.7) | 141 (4.5)  775 (24.8)  1042 (33.4)  1165 (37.3) | <.001 | 2366 (29.2)  2797 (34.5)  1540 (19.0)  1406 (17.3) |
| **Current^c^ need of SABA:**  None  ≤ 2 days/week  > 2 days/week  Everyday | 2486 (70.5)  858 (24.3)  86 (2.4)  94 (2.7) | 563 (38.5)  517 (35.4)  223 (15.3)  159 (10.9) | 547 (17.5)  692 (22.2)  799 (25.6)  1085 (34.7) | <.001 | 3596 (44.3)  2067 (25.5)  1108 (13.7)  1338 (16.5) |
| **Severe exacerbations^d^, n (%)**  1  2  >2 | 315 (8.9)  78 (2.2)  53 (1.5) | 150 (10.3)  42 (2.9)  37 (2.5) | 425 (13.6)  192 (6.1)  217 (6.9) | <.001 | 890 (11.0)  312 (3.8)  3017 (3.8) |
| **Severe exacerbations^d^, mean (SD)** | 0.19 (0.6) | 0.26 (0.8) | 0.59 (1.4) | <.001 | 0.36 (1.1) |
| **Patients participation in asthma education program:**  Yes, last year  Yes, ever  No | 276 (7.8)  755 (21.4)  2495 (70.8) | 140 (9.6)  293 (20.0)  1029 (70.4) | 312 (10.0)  644 (20.6)  2167 (69.4) | 0.028 | 728 (9.0)  1692 (20.9)  5691 (70.1) |
| **Investigators participation in asthma education program:**  Yes, last year  Yes, ever  No | 742 (21.1)  1642 (46.6)  1142 (32.4) | 360 (24.6)  596 (40.8)  506 (34.6) | 760 (24.3)  1118 (35.8)  1245 (39.9) | <.001 | 1862 (23.0)  3356 (41.4)  2893 (35.7) |
| **Patients regularly measuring PEF:**  Yes  No  Sometimes  Never | 169 (4.8)  1878 (53.3)  330 (9.4)  1149 (32.5) | 75 (5.1)  808 (55.3)  129 (8.8)  450 (30.8) | 158 (5.1)  1770 (56.7)  278 (8.9)  917 (29.4) | 0.133 | 402 (5.0)  4456 (54.9)  737 (9.1)  2516 (31.0) |

FeNO: fractional exhaled nitric oxide; GINA: Global Initiative for Asthma; IgE: immunoglobulin E; n: number of patients; PEF: peak expiratory flow; RAST: radioallergosorbent test; SABA: short-acting beta agonist; SD: standard deviation.

^a^ Age at diagnosis < 12 years

^b^ Patients could have done more than one investigation

^c^ Referred to the last week

^d^ Referred to the last 12 months

* p-value Chi−square for categorical variables and Kruskal-Wallis test for quantitative variables

**Table S2:** Lung function

|  | **Controlled**  **n=3526** | **Partly controlled**  **n=1462** | **Uncontrolled**  **n=3123** | **Overall p-value*** | **Overall population**  **n=8111** |
| --- | --- | --- | --- | --- | --- |
| **Spirometry within the last 12 weeks, n (%)** | 2646 (75.0) | 1095 (74.9) | 2205 (70.6) | <.001 | 5946 (73.3) |
| **PEF [L/sec), mean (SD)** | 6.72 (2.23) | 6.20 (2.12) | 5.43 (2.14) | <.001 | 6.13 (2.25) |
| **FEV_1_ [L], mean (SD)** | 2.77 (0.95) | 2.61 (0.91) | 2.29 (0.94) | <.001 | 2.56 (0.96) |
| **FVC [L], mean (SD)** | 3.64 (1.14) | 3.48 (1.12) | 3.12 (1.17) | <.001 | 3.42 (1.17) |
| **FEV_1_ [% predicted normal], mean (SD)** | 88.04 (19.6) | 82.92 (20.9) | 76.03 (23.6) | <.001 | 82.8 (22.0) |
| **FEV_1_/FVC [(%], mean (SD)** | 76.15 (10.25) | 74.96 (12.37) | 73.37 (14.42) | <.001 | 74.90 (12.40) |

FEV_1_: forced expiratory volume in 1 second; FVC: forced vital capacity; L: litre; n: number of patients; PEF: peak expiratory flow; SD: standard deviation.

* p-value based on Chi−square for categorical variables and Kruskal-Wallis test for quantitative variables

**Table S3:** Propensity to adhere to therapy

|  | **Controlled**  **n=3472** | **Partly controlled**  **n=1442** | **Uncontrolled**  **n=3070** | **Overall**  **p-value*** | **Overall population**  **n=7984** |
| --- | --- | --- | --- | --- | --- |
| **Morisky score, mean (SD)** | 1.03 (1.14) | 1.08 (1.16) | 1.08 (1.22) | 0.417 | 1.06 (1.18) |
| **Adherence level, n (%)**  High adherence  Medium adherence  Low adherence | 1541 (44.4)  1479 (42.6)  452 (13.0) | 606 (42.0)  624 (43.3)  212 (14.7) | 1391 (45.3)  1180 (38.4)  499 (16.3) | 0.398 | 3538 (44.3)  3283 (41.1)  1163 (14.6) |

n=number of patients; SD=standard deviation

*p−value based on Kruskal−Wallis test and Gamma Test of Monotone Trend

**Table S4:** Healthcare resources consumption

|  | **Controlled**  **n=3524** | **Partly controlled**  **n=1462** | **Uncontrolled**  **n=3122** | **Overall**  **p-value*** | **Overall population**  **n=8108** |
| --- | --- | --- | --- | --- | --- |
| **Patients with ER attendance^a^, n (%):**  None  1  >1 | 136 (3.9)  3388 (96.1)  92 (2.6)  44 (1.2) | 68 (4.7)  1394 (95.3)  49 (3.4)  19 (1.3) | 237 (7.6)  2885 (92.4)  121 (3.9)  116 (3.7) | <.001  <.001 | 441 (5.4)  7667 (94.6)  262 (3.2)  179 (2.2) |
| **Patients with hospitalization^a^, n (%):**  None  1  >1 | 103 (2.9)  3517 (99.8)  5 (0.1)  2 (0.1) | 43 (2.9)  1459 (99.8)  2 (0.1)  1 (0.1) | 225 (7.2)  3111 (99.6)  6 (0.2)  5 (0.2) | <.001  0.734 | 351 (4.6)  8087 (99.7)  13 (0.2)  8 (0.1) |
| **Days of hospitalization^a,b^, mean (SD)** | 4.3 (5.5) | 4.4 (7.5) | 5.9 (8.2) | 0.031 | 5.3 (7.5) |
| **Number of days^c^ missed from work due to asthma, mean (SD)** | 0.16 (1.6) | 0.39 (3.5) | 1.03 (6.5) | <.001 | 0.53 (4.4) |
| **Patients receiving support by the family, n (%)** | 1342 (38.5) | 646 (45.0) | 1547 (49.5) | <.001 | 3535 (44.4) |

n=number of patients; ER=Emergency Room; SD=standard deviation

^a^ in the last 12 months

^b^ only patients reporting an hospitalization were considered

^c^ in the last 12 weeks

*p-value based on Chi−square (or Fisher exact test) for categorical variables and Kruskal-Wallis test for quantitative variables

**Table S5: Healthcare and economic resources consumption by country**

|  |  | | **Austria** | | **Belgium** | | **France** | | **Germany** | | **Greece** | | **Hungary** | | |
| --- | --- | --- | --- | --- | --- | --- | --- | --- | --- | --- | --- | --- | --- | --- | --- |
|  |  | | **C** | **NWC** | **C** | **NWC** | **C** | **NWC** | **C** | **NWC** | **C** | **NWC** | **C** | | **NWC** |
| **Subjects, n** | | | 157 | 167 | 190 | 192 | 235 | 618 | 568 | 503 | 120 | 219 | 193 | | 658 |
| **Visits to a respiratory specialist^a^** | | | 2.9 (2.5) | 4.0 (4.3) | 2.1 (2.2) | 3.2 (5.2) | 1.2 (1.0) | 1.6 (2.1) | 2.5 (1.8) | 2.5 (1.7) | 4.6 (3.2) | 3.5 (3.3) | 2.3 (1.3) | | 2.2 (1.5) |
| p-value* | |  | 0.078 | | 0.574 | | 0.021 | | 0.846 | | <0.001 | | 0.454 | | |
| **Spirometries** | | | 2.7 (2.4) | 3.9 (3.6) | 1.2 (0.5) | 1.6 (0.9) | 1.4 (0.9) | 1.9 (1.7) | 1.9 (0.9) | 2.0 (1.1) | 3.1 (2.3) | 3.0 (4.3) | 1.8 (1.2) | 2.4 (1.6) | |
| p-value* | |  | 0.006 | | <0.001 | | <0.001 | | 0.078 | | <0.001 | | <0.001 | | |
| **Allergic tests** | | | 1.1 (0.3) | 1.3 (0.7) | 1.2 (0.6) | 1.2 (0.4) | 2.4 (3.3) | 2.0 (3.0) | 2.5 (5.6) | 2.0 (4.7) | 2.6 (6.4) | 4.4 (7.5) | 1.0 (0.0) | 1.2 (0.4) | |
| p-value* | |  | 0.027 | | 0.987 | | 0.775 | | 0.422 | | 0.086 | | 0.294 | | |
| **Days of hospitalization^a^** | | | 0.1 (0.7) | 1.1 (0.3) | 0.1 (0.8) | 0.6 (3.9) | 0.0 (0.5) | 0.0 (0.4) | 0.0 (0.2) | 0.1 (1.1) | 0.3 (1.8) | 0.8 (2.9) | 0.3 (2.6) | 0.8 (4.6) | |
| p-value* | |  | 0.653 | | 0.007 | | 0.204 | | 0.135 | | 0.215 | | 0.126 | | |
| **Single day/emergency room access^a^** | | | 0.0 (0.1) | 0.0 (0.1) | 0.0 (0.1) | 0.1 (0.4) | 0.0 (0.1) | 0.0 (0.2) | 0.0 (0.1) | 0.0 (0.2) | 0.2 (0.6) | 0.3 (1.0) | 0.0 (0.0) | 0.0 (0.1) | |
| p-value* | |  | 0.969 | | 0.001 | | 0.317 | | 0.143 | | 0.616 | | 0.348 | | |
| **Days missed from work due to asthma^b^** | | | 0.1 (0.6) | 0.6 (2.6) | 0.0 (0.4) | 0.7 (3.4) | 0.1 (0.7) | 0.6 (6.5) | 0.0 (0.1) | 0.7 (4.9) | 0.8 (3.6) | 0.9 (3.2) | 0.0 (0.4) | 0.2 (1.8) | |
| p-value* | |  | 0.014 | | 0.002 | | 0.114 | | <0.001 | | 0.480 | | 0.488 | | |
| **Support received by the family, n (%)** | | | 49 (31.2) | 66 (39.5) | 34 (17.9) | 87 (45.3) | 51 (21.7) | 383 (62.0) | 108 (19.0) | 153 (30.4) | 81 (67.5) | 169 (77.2) | 59 (30.6) | 133 (20.2) | |
| p-value** | |  | 0.118 | | <.001 | | <.001 | | <.001 | | 0.053 | | 0.002 | | |
| **Investigators participation in education program^a^, n (%)** | | | 17 (10.8) | 4 (2.4) | 35 (58.3) | 27 (37.0) | 5 (7.1) | 4 (16.7) | 7 (1.3) | 18 (3.6) | 39 (52.7) | 95 (51.4) | 137 (71.7) | 359 (73.3) | |
| p-value** | |  | 0.002 | | 0.014 | | 0.171 | | 0.011 | | 0.844 | | 0.685 | | |

|  |  | **Italy** | | **Netherlands** | | **Poland** | | **Spain** | | **Turkey** | | **United Kingdom** | |
| --- | --- | --- | --- | --- | --- | --- | --- | --- | --- | --- | --- | --- | --- |
|  |  | **C** | **NWC** | **C** | **NWC** | **C** | **NWC** | **C** | **NWC** | **C** | **NWC** | **C** | **NWC** |
| **Subjects, n** | | 684 | 417 | 73 | 201 | 321 | 716 | 611 | 430 | 215 | 224 | 159 | 240 |
| **Visits to a respiratory specialist^a^** | | 1.5 (1.5) | 2.3 (3.2) | 2.5 (1.7) | 3.3 (2.4) | 1.1 (1.9) | 1.3 (2.3) | 1.8 (1.7) | 2.6 (4.8) | 2.4 (3.3) | 2.8 (3.9) | 0.1 (0.4) | 0.1 (0.7) |
| p-value* |  | 0.002 | | 0.005 | | 0.138 | | <0.001 | | 0.303 | | 0.995 | |
| **Spirometries** | | 2.9 (4.8) | 2.5 (5.2) | 2.7 (2.4) | 2.7 (2.6) | 1.5 (1.0) | 1.5 (1.0) | 1.3 (0.9) | 1.5 (1.9) | 2.1 (1.80) | 2.1 (2.2) | 1.4 (1.3) | 1.1 (0.5) |
| p-value* |  | 0.118 | | 0.451 | | 0.246 | | 0.126 | | 0.256 | | 0.646 | |
| **Allergic test** | | 1.1 (0.4) | 1.7 (2.4) | 1.1 (0.4) | 1.2 (0.4) | 2.3 (4.2) | 1.5 (2.1) | 1.0 (0.0) | 1.3 (1.8) | 1.0 (0.1) | 1.1 (0.3) | 0 | 1.7 (1.5) |
| p-value* |  | 0.093 | | 0.731 | | 0.370 | | 0.333 | | 0.177 | | - | |
| **Days of hospitalization^a^** | | 0.1(0.8) | 0.3 (2.1) | 0.4 (1.4) | 0.6 (2.1) | 0.1 (0.7) | 0.2 (1.5) | 0.3 (1.9) | 0.3 (1.5) | 0.0 (0.2) | 0.3 (1.4) | 0.1 (0.5) | 0.1 (1.1) |
| p-value* |  | 0.007 | | 0.341 | | 0.003 | | <0.001 | | <0.001 | | 0.190 | |
| **Single day/emergency room access^a^** | | 0.0 (0.2) | 0.2 (1.0) | 0.1 (0.2) | 0.1 (0.5) | 0.0 (0.2) | 0.0 (0.2) | 0.2 (0.6) | 0.6 (1.6) | 0.2 (1.0) | 0.9 (2.3) | 0.0 (0.2) | 0.0 (0.3) |
| p-value* |  | 0.001 | | 0.355 | | 0.284 | | <0.001 | | <0.001 | | 0.565 | |
| **Days missed from work due to asthma^b^** | | 0.2 (1.5) | 0.9 (4.6) | 0.5 (3.5) | 1.7 (7.6) | 0.1 (1.7) | 0.8 (5.3) | 0.3 (2.3) | 2.1 (11.4) | 0.1 (0.9) | 1.1 (5.1) | 0.1 (0.6) | 0.4 (2.3) |
| p-value* |  | <0.001 | | 0.038 | | <0.001 | | <0.001 | | <0.001 | | 0.016 | |
| **Support received by the family, n (%)** | | 263 (38.5) | 160(38.4) | 49 (67.1) | 113 (56.2) | 210 (65.4) | 504 (70.4) | 256 (41.9) | 250 (58.1) | 177 (82.3) | 159 (71.0) | 5 (3.1) | 16 (6.7) |
| p-value** |  | 0.979 | | 0.053 | | 0.110 | | <0.001 | | 0.005 | | 0.123 | |
| **Investigators participation in education program^a^, n (%)** | | 105 (41.8) | 27 (32.5) | 2 (11.1) | 8 (15.4) | 92 (36.4) | 370 (59.6) | 197 (41.8) | 96 (33.2) | 66 (42.9) | 66 (40.0) | 40 (30.1) | 46 (23.8) |
| p-value** |  | 0.133 | | 0.655 | | <0.001 | | 0.018 | | 0.605 | | 0.209 | |

C: patients with controlled asthma (6-item ACQ score < 1.5); NWC: patients with not well controlled asthma (6-item ACQ <1.5).

n: number of patients. For some variables the n could be slightly different from those reported in the first row because of missingness.

Data are presented as mean (SD) unless otherwise stated;* p-value based on Chi−square test; ** p-value based on Wilcoxon test.

^a^ in the last 12 months; ^b^ in the last three months
